# Ações intersetoriais e o reconhecimento de uma fonte de cuidado da atenção primária por adolescentes brasileiros

**DOI:** 10.1590/0102-311XPT195923

**Published:** 2024-11-04

**Authors:** Maísa Mônica Flores Martins, Nília Maria de Brito Lima Prado, Leila Denise Alves Ferreira Amorim, Ana Luiza Queiroz Vilasbôas, Rosana Aquino

**Affiliations:** 1 Instituto de Saúde Coletiva, Universidade Federal da Bahia, Salvador, Brasil.; 2 Escola Bahiana de Medicina e Saúde Pública, Salvador, Brasil.; 3 Instituto Multidisciplinar em Saúde, Universidade Federal da Bahia, Vitória da Conquista, Brasil.; 4 Instituto de Matemática, Universidade Federal da Bahia, Salvador, Brasil.

**Keywords:** Serviços de Saúde, Atenção Primária à Saúde, Estratégia Saúde da Família, Ação Intersetorial, Health Services, Primary Health Care, Family Health Strategy, Intersectoral Collaboration, Servicios de Salud, Atención Primaria de Salud, Estrategia de Salud Familiar, Acción Intersectorial

## Abstract

Este estudo teve como objetivo analisar a associação entre o desenvolvimento de ações intersetoriais entre escola/serviços de saúde da atenção primária à saúde (APS) e o reconhecimento de uma fonte usual do cuidado de APS entre adolescentes brasileiros. Trata-se de um estudo transversal, a partir da *Pesquisa Nacional de Saúde do Escolar* de 2015, realizado com 97.903 adolescentes, com amostragem complexa. A associação entre ações intersetoriais entre serviços de APS e escolas e o reconhecimento da fonte usual do cuidado da APS foram estimadas por meio da razão de prevalência (RP), com uso do modelo de regressão logística, sendo considerado o fator de ponderação amostral, por meio do Stata 14.0 Dos adolescentes analisados, 72,8% estudavam em escolas que desenvolviam ações intersetoriais com os serviços de APS. Entre esses, observou-se associação entre o reconhecimento da fonte usual do cuidado da APS e ações intersetoriais (RP = 1,11; IC95%: 1,08-1,14). Quando analisado para ações do Programa Saúde na Escola (RP = 1,40; IC95%: 1,37-1,43) e o desenvolvimento de ações entre a escola e os serviços de APS (RP = 1,08; IC95%: 1,05-1,12). Os resultados mostram que existe uma associação positiva entre o reconhecimento dos serviços de APS como uma fonte usual do cuidado e as ações intersetoriais. Entretanto, existem desafios na articulação entre os setores de saúde e educação, na perspectiva de uma prática que se configura como intersetorial, para a implementação das ações de prevenção e de promoção da saúde ao adolescente na escola. Envolvem, ainda, maior conhecimento sobre a percepção dos adolescentes sobre a qualidade do serviço ofertado pelas unidades de saúde.

## Abstract (en)

This study aimed to analyze the association between the development of intersectoral actions between school/primary health care (PHC) services and the recognition of a usual source of care of PHC among Brazilian adolescents. This is a cross-sectional study, from the *Brazilian National Survey of School Health* (2015) conducted with a complex sampling of 97,903 adolescents. The association between intersectoral actions between PHC services and schools and the recognition of a usual source of care of PHC were estimated by the prevalence ratio (PR), using the logistic regression model and considering the sample weight factor with Stata 14.0 Of the analyzed adolescents, 72.8% of them studied in schools that developed intersectoral actions with PHC services. Among adolescent students from schools that developed intersectoral actions, an association was observed between the recognition of usual source of care of PHC and intersectoral actions (PR = 1.11; 95%CI: 1.08-1.14). When analyzed for actions of the School Health Program (PR = 1.40; 95%CI: 1.37-1.43), and the development of actions between the school and PHC services (PR = 1.08; 95%CI: 1.05-1.12). The results show that there is a positive association between the recognition of PHC services as a usual source of care and intersectoral actions. However, from the perspective of an intersectoral practice, there are challenges in the articulation between health and education sectors for implementing prevention and promoting adolescent health in school. They involve greater knowledge about the adolescents’ perception of the quality of the service offered by health units.

## Resumen

Este estudio tuvo como objetivo analizar la asociación entre el desarrollo de acciones intersectoriales entre el colegio/servicios de salud de la atención primaria de salud (APS) y el reconocimiento de un recurso habitual de atención de la APS entre los adolescentes brasileños. Se trata de un estudio transversal, basado en la *Encuesta Nacional de Salud del Escolar* (2015) realizada a 97.903 adolescentes, con muestreo complejo. La asociación entre las acciones intersectoriales entre los colegios/servicios de APS y el reconocimiento de un recurso habitual de atención de la APS se estimaron por la razón de prevalencia (RP), a partir del modelo de regresión logística y del factor de ponderación de la muestra mediante el Stata 14.0 El 72,8% de los adolescentes estudiaban en colegios que desarrollaron acciones intersectoriales con los servicios de APS. Entre los estudiantes de colegios que desarrollaron acciones intersectoriales, se observó una asociación entre el reconocimiento de recurso habitual de atención de APS y las acciones intersectoriales (RP = 1,11; IC95%: 1,08-1,14). En el análisis de las acciones del Programa Salud en la Escuela (RP = 1,40; IC95%: 1,37-1,43), hubo un desarrollo de acciones entre el colegio y los servicios de APS (RP = 1,08; IC95%: 1,05-1,12). Los resultados apuntan a una asociación positiva entre el reconocimiento de los servicios de APS como recurso habitual de atención y las acciones intersectoriales. Sin embargo, se encuentran desafíos en la articulación entre los sectores de salud y educación desde una acción intersectorial para implementar acciones de prevención y promoción de la salud para adolescentes escolares. Esto también implica un mayor conocimiento sobre la percepción de los adolescentes sobre la calidad del servicio que prestan los centros de salud.

## Introdução

Ação intersetorial em saúde pode ser definida como a capacidade de integração entre agentes e distintos setores com capacidades técnicas específicas e que se articulam em espaços de gestão, planejamento, execução e avaliação das necessidades dos sujeitos ^1^. A intersetorialidade se fundamenta em iniciativas que buscam superar a fragmentação dos conhecimentos e das políticas sociais, por meio de práticas e ações integradas de diferentes setores, que se articulam, interagem e se complementam no desenvolvimento de estratégias conjuntas, com a perspectiva de produzir efeitos que possam reduzir as iniquidades em saúde e melhorar a qualidade de vida de populações ^2^ ^,^ ^3^ ^,^ ^4^.

As ações intersotirais em saúde têm sido tema de debates internacionais desde a década de 1970, por meio da publicação da *Declaração de Alma Ata* (Cazaquistão) ^5^. A abordagem das ações intersetoriais em saúde busca identificar e discutir por meio do ideário da intersetorialidade, estratégias políticas para o fortalecimento de ações de promoção da saúde no âmbito da atenção primária à saúde (APS) ^1^, que inclui a organização dos serviços e a garantia do acesso de adolescentes a ações de promoção à saúde, prevenção e atenção a agravos.

No Brasil, o desenvolvimento das ações intersetoriais representa um dos propósitos da Política Nacional de Atenção Básica (PNAB), baseada em um modelo de atenção que visa ao cuidado longitudinal do indivíduo para a maioria de seus problemas e suas necessidades de saúde ^6^ ^,^ ^7^. Entre as atribuições da equipe multiprofissional dos serviços de APS, a implementação de ações articuladas entre diferentes setores, mediante parcerias e recursos da comunidade, pode potencializar essas ações, além de favorecer a integração de projetos orientados para a promoção da saúde ^8^ ^,^ ^9^. Por outro lado, a longitudinalidade, um dos atributos da APS, é conceituada como a existência de uma fonte usual de cuidado, capaz de estabelecer vínculo entre usuários e profissionais ^10^, o que pode favorecer o acompanhamento e a atenção adequada da saúde dos adolescentes ^11^.

No entanto, no Brasil, a atenção integral à saúde dos adolescentes configura-se como uma agenda incompleta, uma vez que diversas iniciativas de políticas públicas têm sido apresentadas, como a Política Nacional de Atenção Integral à Saúde do Adolescente e Jovem, cuja implementação não tem avançado ^12^. Esse cenário condiciona o adolescente a enfrentar obstáculos importantes para receber uma atenção à saúde integral e de qualidade apropriada para sua idade, dada a existência de serviços fragmentados e não alinhados às suas necessidades ^13^.

Como a perspectiva de fortalecer um modelo de cuidado à saúde abrangente, o Programa Saúde na Escola (PSE) foi criado em 2007, com enfoque na execução de atividades de prevenção de doenças e promoção da saúde, em articulação com serviços de APS, especificamente pela Estratégia Saúde da Família (ESF) ^14^. O PSE busca integrar e articular ações intersetoriais, entre saúde e educação, com o objetivo de melhorar a qualidade de vida de adolescentes, estudantes da rede pública de ensino, visando enfrentar as vulnerabilidades nesse período da vida ^15^ ^,^ ^16^.

O programa faz parte das propostas da Política Nacional de Promoção da Saúde (PNPS) ^17^ ^,^ ^18^, sendo uma das políticas públicas de grande destaque para os adolescentes, ao propor ações de avaliação clínica, promoção da saúde, prevenção de doenças e agravos, formação de profissionais da educação e da saúde ^19^, bem como o estabelecimento de uma relação de confiança entre escola, aluno e serviço de saúde ^9^ ^,^ ^20^. Partindo dessa premissa, este estudo tem como hipótese que o desenvolvimento das ações intersetoriais entre os serviços de APS e as escolas pode favorecer uma relação de vínculo e confiabilidade entre sujeitos sociais, a partir da integração entre os setores da saúde e da educação, contribuindo para que os adolescentes reconheçam os serviços de APS adscritos ao território-área, como sua fonte de cuidados; o que, por sua vez, está associado a um maior acesso às ações de educação em saúde, maior uso de serviços preventivos, como vacinação, redução no uso de cuidados de emergência, melhora do estado de saúde, maior satisfação, e aumento da confiança ^21^ ^,^ ^22^ ^,^ ^23^, além de estabelecer a corresponsabilização, o vínculo e um processo de construção compartilhada ^24^.

Sendo assim, este estudo tem como objetivo analisar a associação entre o desenvolvimento de ações intersetoriais entre escola/serviços de saúde da APS e o reconhecimento de uma fonte usual do cuidado de APS entre adolescentes brasileiros.

## Metodologia

Estudo do tipo transversal, considerando dados provenientes da *Pesquisa Nacional de Saúde do Escolar* (PeNSE), realizada no ano 2015, por meio do convênio celebrado entre Ministério da Saúde, Ministério da Educação com apoio do Instituto Brasileiro de Geografia e Estatística (IBGE).

A população do estudo, conforme a nota técnica ^25^ e os manuais oficiais divulgados pelo IBGE, foi constituída por adolescentes matriculados no 9º ano do Ensino Fundamental (antiga 8ª série) de escolas públicas ou privadas, situadas nas áreas urbanas e rurais de todo o território nacional ^26^. No entanto, a partir da análise do banco de dados de domínio público da PeNSE 2015, e da verificação de seu dicionário de códigos, observa-se que a população foi composta por adolescentes/escolares a partir do 6º ano e com idade a partir de 11 anos.

Para a condução deste estudo, foram utilizados dados da amostra 1 da PeNSE 2015, sendo consideradas informações dos indivíduos e das escolas. Todos os alunos presentes no dia da coleta e nas turmas sorteadas foram convidados a participar da pesquisa ^26^. Participaram da terceira edição da PeNSE, em 2015, um total de 102.301 estudantes brasileiros, sendo registrados 102.072 questionários válidos, em 3.040 escolas, 4.159 turmas e 203 estratos ^26^. Para este estudo, utilizou-se a amostra final de 97.903 adolescentes, conforme a definição da variável dependente.

As especificações sobre o processo amostral considerando a estratificação do território e uma amostra por conglomerado estão disponíveis no livro da PeNSE de 2015 ^26^. Segundo essa documentação, para estimar o número de alunos de um plano amostral por conglomerado em estágios e uma seleção com probabilidades proporcionais, considerou-se uma estimativa de proporção de 50%, com margem de erro de 0,03% em valor absoluto com intervalo de 95% de confiança (IC95%), sendo o primeiro estágio da amostragem constituído das escolas, e o segundo estágio, das turmas elegíveis nas escolas selecionadas do Ensino Fundamental ^26^ ^,^ ^27^.

### Variável dependente

A variável dependente do estudo é a declaração do respondente sobre ter uma fonte usual de cuidado de APS, definido, a partir da adaptação do conceito de fonte usual de cuidado de Starfield ^10^, como a existência de um serviço de APS que o adolescente procura quando está doente ou quando necessita de cuidados de saúde. Para construção dessa variável, foram consideradas duas questões da PeNSE (“Nos últimos 12 meses você procurou algum serviço ou profissional de saúde para atendimento relacionado à própria saúde?” e “Nos últimos 12 meses, qual foi o serviço de saúde que você procurou com mais frequência?”), em que os casos nos quais o adolescente afirmou ter procurado um serviço de APS com maior frequência são tidos como “ter uma fonte usual de cuidado de APS”.

### Variáveis independentes principais

Foram utilizadas três variáveis independentes principais dicotômicas (sim/não), a saber: ações intersetoriais; ações do PSE; e ações desenvolvidas entre escola/serviços de saúde:

(1) A variável referente à existência de ações intersetoriais em saúde foi definida a partir de três perguntas do inquérito que visavam identificar a adesão e a implantação do PSE ou a presença de uma relação entre a escola e os serviços da APS (“A escola aderiu ao Programa Saúde da Escola (PSE)?”, “A escola implementa ações do PSE?” e “A escola realiza ações conjuntas com Unidade Básica de Saúde ou Equipe de Saúde da Família ou Equipe de Atenção Básica?”), sendo considerado como afirmativo os casos em que os responsáveis pelas escolas disseram que a instituição aderiu e implantou as ações do PSE, ou realizou ações conjuntas com os serviços de APS;

(2) A existência de ações do PSE foi definida como afirmativa para aqueles adolescentes matriculados em escolas que aderiram e implantaram as ações do PSE, incluindo na categoria “sim” da variável apenas os adolescentes de escolas públicas, conforme a definição do programa ^28^;

(3) A variável ações desenvolvidas entre escola e serviços de APS foi definida a partir da pergunta que considerava ações conjuntas da escola e de serviços de APS (“A escola realiza ações conjuntas com Unidade Básica de Saúde ou Equipe de Saúde da Família ou Equipe de Atenção Básica?”).

Vale destacar que, para a análise das ações intersetoriais por meio da variável ações do PSE, consideraram-se dados de 94.952 adolescentes que tinham informação sobre a adesão e a implementação de ações do PSE, de modo que se considerou dados dos adolescentes das escolas públicas de ensino (categoria “sim”), dos adolescentes de escolas privadas que aderiram ao PSE e dos demais adolescentes oriundos das escolas que não aderiram (categoria “não”).

Para a análise da variável ações desenvolvidas entre escola e serviço de APS, exclui-se da análise aqueles indivíduos cujas escolas informaram ter ações de PSE, sendo avaliados 58.588 adolescentes.

### Covariáveis

Foram definidas como variáveis de ajustes deste estudo: região de residência (Nordeste/Norte e Sudeste/Sul/Centro-oeste), tipo de município (capital e não-capital), zona de localização da escola (rural e urbana) e dependência administrativa da escola (pública e privada). A seleção dessas variáveis ocorreu para além de critérios estatísticos.

Foi realizada uma análise descritiva por meio do teste χ^2^ de Pearson entre o desenvolvimento das ações intersetoriais e as variáveis relacionadas à identificação das escolas (região de residência; tipo de município; localização da escola; e dependência administrativa). Essas análises bivariadas foram calculadas por meio do teste χ^2^ de Pearson, com correção de Rao-Scott de segunda ordem para o desenho amostral ^29^ ^,^ ^30^ considerando a estrutura de conglomerados dos dados. A análise foi realizada com base no comando *svy*: *tabulate* do software Stata 14.0 (https://www.stata.com), sendo considerado um nível de 5% de significância.

A magnitude da associação entre o reconhecimento de uma fonte usual de cuidado da APS e as ações intersetoriais foi estimada pela razão de prevalência (RP) e seus respectivos IC95%, por meio de um modelo de regressão logística. Posteriormente, metodologia similar foi utilizada para estimar a associação entre fonte usual de cuidado de APS e as demais variáveis independentes principais: ações do PSE e ações entre a escola e serviços de APS.

Durante o processo de modelagem, foi realizada análise de colinearidade. Após essa etapa, todas as variáveis pré-selecionadas foram incluídas no modelo. A comparação entre modelos foi feita com uso dos critérios de informações bayesiana, de Akaike e Schwarz.

As RP foram estimadas pelos modelos de regressão logística, por meio do uso do pacote *adjrr* do software Stata 14.0. Todas as análises foram realizadas usando Stata 14.0, e para as devidas adequações e correções para um estudo de amostragem complexa, considerou-se o efeito dos conglomerados (escolas), sendo utilizado o fator de ponderação amostral, em função das condições do processo de amostragem ^27^.

A PeNSE 2015 foi aprovada na Comissão Nacional de Ética em Pesquisa (CONEP), do Conselho Nacional de Saúde, que regulamenta e aprova pesquisas em saúde envolvendo seres humanos (parecer Conep nº 1.006.467, de 30 de março de 2015). Este estudo, por tratar de dados de domínio público, dispensa a submissão para apreciação de um comitê de ética.

## Resultados

A maioria dos adolescentes (72,8%) estudava em escolas que desenvolviam ações intersetoriais com os serviços de APS, sendo observados maiores percentuais nas regiões Nordeste (79,2%) e Norte (77,5%), em municípios considerados não-capitais (76%), localizados na zona rural (89,2%) e em escolas públicas (80,6%) (Tabela 1).

**Tabela 1:** Distribuição dos adolescentes e características das escolas segundo desenvolvimento de ações intersetoriais. *Pesquisa Nacional de Saúde do Escolar* de 2015, Brasil.

Características	n	Ações intersetoriais em saúde (%)	Valor de p
Total	97.903	72,8	
Região de residência			0,000
Norte	22.880	77,5	
Nordeste	34.783	79,2	
Sudeste	17.244	68,5	
Sul	9.383	74,8	
Centro-oeste	13.613	77,4	
Tipo de município			0,001
Capital	49.096	66,2	
Não-capital	48.807	76,0	
Localização da escola			0,000
Urbana	89.936	72,4	
Rural	7.967	89,2	
Dependência administrativa			0,000
Pública	77.332	80,6	
Privada	20.571	34,5	

Fonte: *Pesquisa Nacional de Saúde do Escolar* de 2015 ^26^.

Na análise do modelo de regressão logística entre o reconhecimento da fonte usual de cuidado de APS e o desenvolvimento de ações intersetoriais, observou-se diferenças estatisticamente significativas para o modelo multivariado (RP = 1,11; IC95%: 1,08-1,14). Entre as variáveis de ajustes que apresentaram associações positivas no modelo multivariado, destacam-se: residir no Nordeste/Norte e ser estudante de escola pública (Tabela 2).

**Tabela 2:** Razão de prevalência (RP) multivariada por meio do modelo de regressão logística para a associação entre o desenvolvimento de ações intersetoriais e o reconhecimento de uma fonte usual de cuidado de atenção primária à saúde (APS). *Pesquisa Nacional de Saúde do Escolar* de 2015, Brasil.

Variáveis	Modelo multivariado (fonte usual de cuidado de APS)	
	RP	IC95%
Ações intersetoriais	1,11	1,08-1,14
Região de residência		
Nordeste/Norte	1,09	1,06-1,11
Tipo de município		
Capital	0,86	0,84-0,88
Zona de localização da escola		
Urbano	0,87	0,84-0,91
Dependência administrativa da escola		
Pública	2,98	2,84-3,13

IC95%: intervalo de 95% de confiança.

Fonte: *Pesquisa Nacional de Saúde do Escolar* de 2015 ^26^.

Quanto à análise entre o reconhecimento da fonte usual de cuidado de APS e o desenvolvimento de ações do PSE, observou-se diferenças estatisticamente significativas para o modelo multivariado (RP = 1,40; IC95%: 1,37-1,43). Entre as variáveis de ajustes, foram encontradas associações positivas apenas para a variável residir no Nordeste/Norte. As variáveis restantes apresentaram associações negativas (Tabela 3).

**Tabela 3:** Razão de prevalência (RP) multivariada por meio do modelo de regressão logística para a associação entre o desenvolvimento de ações pelo Programa Saúde na Escola (PSE) e o reconhecimento de uma fonte usual de cuidado de atenção primária à saúde (APS). *Pesquisa Nacional de Saúde do Escolar* de 2015, Brasil.

Variáveis	Modelo multivariado (fonte usual de cuidado de APS)	
	RP	IC95%
PSE	1,40	1,37-1,43
Região de residência		
Nordeste/Norte	1,08	1,05-1,10
Tipo de município		
Capital	0,78	0,76-0,80
Zona de localização da escola		
Urbano	0,79	0,76-0,82

IC95%: intervalo de 95% de confiança.

Fonte: *Pesquisa Nacional de Saúde do Escolar* de 2015 ^26^.

Quanto à análise de associação entre o reconhecimento de uma fonte usual de cuidado de APS e o desenvolvimento das ações de saúde entre a escola e os serviços de APS, foi observada associação positiva com diferença estatisticamente significativa para o modelo multivariado (RP = 1,08; IC95%: 1,05-1,12). Entre as variáveis de ajustes, observou-se associações positivas para os adolescentes residentes no Nordeste/Norte e estudantes de escolas públicas. As demais variáveis apresentam associações negativas (Tabela 4).

**Tabela 4:** Razão de prevalência (RP) multivariada por meio do modelo de regressão logística para a associação entre ações intersetoriais desenvolvidas entre a escola e os serviços de atenção primária à saúde (APS) e o reconhecimento de uma fonte usual de cuidado de APS. *Pesquisa Nacional de Saúde do Escolar* de 2015, Brasil.

Variáveis	Modelo multivariado (fonte usual de cuidado de APS)	
	RP	IC95%
Ações desenvolvidas entre a escola e a UBS	1,08	1,05-1,12
Região de residência		
Nordeste/Norte	1,10	1,06-1,13
Tipo de município		
Capital	0,85	0,82-0,88
Zona de localização da escola		
Urbano	0,89	0,84-0,94
Dependência administrativa da escola		
Pública	2,97	2,83-3,13

IC95%: intervalo de 95% de confiança; UBS: unidade básica de saúde.

Fonte: *Pesquisa Nacional de Saúde do Escolar* de 2015 ^26^.

## Discussão

Os resultados deste estudo ratificam que estudantes brasileiros oriundos de instituições de ensino que desenvolveram ações intersetoriais entre os setores saúde e educação reconhecem um serviço de APS como sua fonte usual de cuidado. O estudo mostrou que há associação entre o reconhecimento da fonte usual de cuidado de APS e as ações intersetoriais em saúde, analisado pelo indicador construído e pelas variáveis independentes principais: PSE e ações entre escolas e serviços de APS. Diferenças mais acentuadas foram observadas com respeito às ações do PSE.

Na literatura científica disponível até o momento, não há evidências de outras pesquisas que tenham proposto uma análise dessa natureza para as ações intersetoriais, tampouco para avaliar sua relação com uma fonte usual de cuidado para os adolescentes.

No cenário internacional, especialmente em países em que os adolescentes estão inseridos em contextos de maior conflito, seja por violência ou por questões humanitárias, a avaliação de programas direcionados para esse público aponta que essas iniciativas proporcionam acesso à saúde, meios de geração de renda e lazer. A investigação sobre os resultados desses programas é de grande relevância para o rompimento das iniquidades ^31^.

Neste estudo, verifica-se que os adolescentes residentes nas regiões Norte/Nordeste com consideráveis desigualdades econômicas e sociais, além de estarem mais susceptíveis às vulnerabilidades em saúde ^32^, apresentam maior probabilidade de reconhecerem a APS como sua fonte usual de cuidado. Esse panorama demonstra a real necessidade de estudos com essa parcela da população, buscando traçar medidas de intervenção para modificar tal realidade. Diante desse cenário, vale ressaltar que a disposição de uma fonte usual de cuidado, além de um importante indicador de acesso e disponibilidade de serviços ^33^, demonstra que as coberturas mais homogêneas da APS/ESF na Região Nordeste podem refletir no reconhecimento do adolescente da APS como sua fonte usual de cuidado ^34^.

É importante destacar que a Organização Mundial da Saúde (OMS) tem reforçado iniciativas com o propósito de sensibilizar os serviços de saúde a acolher e atender os adolescentes de forma adequada, considerando as reais necessidades e implementando políticas públicas que sejam compartilhadas de forma intersetorial, entre a escola e os serviços de saúde ^35^. As escolas promotoras de saúde basearam as ações de promoção na participação e na construção coletiva, no empoderamento e na autonomia dos sujeitos, buscando reverter o caráter biomédico e assistencialista dos programas de saúde escolar, revendo o posicionamento e a prática do setor saúde no âmbito da educação, especialmente na condução do planejamento das ações ^36^. As iniciativas preconizam o envolvimento de forma efetiva da comunidade escolar para o desenvolvimento de ações de promoção da saúde, um fator de extrema relevância para capacitar professores e facilitar o acesso desse público aos serviços de saúde ^37^.

No Brasil, entre as estratégias de ações intersetoriais, destaca-se o PSE, que busca contribuir no desenvolvimento de ações de prevenção, promoção e atenção à saúde da criança e do adolescente estudante de escolas públicas ^28^. Neste estudo, observa-se uma probabilidade 34% maior no reconhecimento da fonte usual de cuidado APS entre os adolescentes estudantes de escolas que aderiram e implantaram ações do PSE, comparado aos que não desenvolvem ações do PSE. Esse programa tem a finalidade de articular intervenções do Sistema Único de Saúde (SUS) às ações de educação da rede pública, contribuindo com a construção de uma atenção social, com foco na construção da cidadania e do respeito pelos direitos humanos, no enfrentamento das vulnerabilidades no campo da saúde e no desenvolvimento escolar, além de promover a intercomunicação entre saúde e educação ^38^ ^,^ ^39^.

O PSE busca a integração das práticas no âmbito das escolas e das unidades de APS por meio da ESF, partindo da lógica do cuidado em saúde ^37^. Conforme a proposta do PSE, o componente de promoção da saúde e prevenção de doenças e agravos, possibilita o desenvolvimento de diferentes tipos de ações, de caráter intersetorial, o qual propõe condições educativas importantes para adolescentes, por meio de metodologias participativas de aprendizagem, além de assegurar o compartilhamento de conhecimentos e o desenvolvimento de escolhas mais positivas para a saúde, favorecendo, assim, o protagonismo dos estudantes para o autocuidado ^40^. Vale destacar que a execução dessas atividades deve ser planejada e implementada por profissionais das equipes da APS e da educação ^19^.

Ademais, o PSE preconiza a avaliação clínica dos estudantes e ações de promoção e prevenção de doenças e agravos, tendo em vista que os adolescentes que permanecem na escola têm maior probabilidade de procura de serviços de saúde para cuidados preventivos, bem como para cuidados de condições de saúde específicas. O aumento dessas ações do PSE desenvolvidas pelos profissionais das equipes da ESF é fruto do desempenho do setor saúde dos municípios, por meio da melhoria do planejamento e da articulação das ações de saúde juntos às escolas, com a perspectiva de diagnosticar os reais problemas e as necessidades de saúde, favorecendo, assim, o reconhecimento de uma fonte usual de cuidado de APS e, consequentemente, o acesso do estudante à rede de atenção à saúde ^32^ ^,^ ^41^.

Todavia, estudos que abordam a temática das ações intersetoriais, especialmente do PSE, têm destacado um alto investimento dos profissionais responsáveis pelo programa em ações de caráter clínico e psicossocial ^14^ ^,^ ^42^ ^,^ ^43^ ^,^ ^44^, sendo relacionado a possíveis influências do modelo biomédico de atenção à saúde, que tem como perspectiva uma atenção individual, biologicista e fragmentada ^42^ ^,^ ^45^ ^,^ ^46^. Apesar dessa tendência nas ações conduzidas pelo PSE, vale destacar que mesmo as ações de caráter clínico podem subsidiar o desenvolvimento de ações que visem uma prevenção secundária, a exemplo de prevenção de problemas visuais e tratamento oportuno para as crianças e adolescentes ^47^.

Além de questões relacionadas à implementação das ações intersetoriais voltadas para o público em questão, é preciso considerar fatores econômicos e sociais que podem determinar as condições de vida e saúde desses adolescentes, bem como influenciar no reconhecimento de uma fonte usual de cuidado de APS. As desigualdades socioeconômicas e relativas a situações de risco foram associadas ao reconhecimento de uma fonte usual de cuidado de APS. Diferenças marcantes foram observadas para o tipo de dependência administrativa, sendo uma prevalência em torno de três vezes maior entre estudantes de escolas públicas nas quais houve desenvolvimento de ações intersetoriais em saúde entre os setores de saúde e educação.

Em face da discussão apresentada, vale ponderar algumas limitações para a interpretação dos achados. A princípio, uma das limitações do estudo deve-se ao desenho de corte transversal, não podendo ser estabelecida inferência causal entre as ações intersetoriais e o reconhecimento da fonte usual de cuidado de APS, sendo necessária uma interpretação cautelosa dos resultados. Além disso, é importante considerar a definição da variável dependente - fonte usual de cuidado de APS - que se baseou como pergunta do inquérito e o serviço de saúde que utilizou com mais frequência, não sendo possível avaliar a diferenças entre ter uma fonte usual de cuidado, serviço de saúde, ou o mesmo médico/profissional de saúde que atua naquele serviço. Por fim, vale ressaltar o tipo de coleta de dados, por meio do autorrelato, o que pode representar uma superestimação dos dados reais do reconhecimento de uma fonte usual de cuidado. Essas lacunas podem ser exploradas em pesquisas com outras abordagens metodológicas.

Apesar das limitações, o estudo apresenta potencialidades devido à escassez de estudos sobre a temática e da abordagem quantitativa, o que o distingue da maioria das investigações nacionais com adolescentes, por sua natureza de inquérito escolar com a possibilidade de levantamento dos fatores associados ao reconhecimento de uma fonte de cuidados e de ações intersetoriais entre saúde e educação para uma amostra representativa dos adolescentes do território nacional. Vale ainda destacar que, ao utilizar dados agregados do banco da PeNSE para a definição da variável independente principal - ações intersetoriais -, limitou-se a exploração e a definição das variáveis de confundimento, pois, além de não haver muitas variáveis para exploração no banco da PeNSE, não foi possível utilizar dados de outras fontes devido à indisponibilidade das informações dos municípios não capitais, sendo inviável o merge entre os bancos.

## Considerações finais

Este estudo observou que, apesar da elevada frequência de adolescentes brasileiros estudantes de escolas que desenvolveram ações intersetoriais, seja por meio do PSE ou de outras relações entre os serviços de saúde da APS e educação, o PSE, como uma política pública, demonstrou importante relevância para os estudantes reconhecerem os serviços de APS como sua fonte usual de cuidado. Nesse sentido, sugere-se realizar novos estudos quantitativos, com diferentes abordagens metodológicas, que abordem a temática central deste estudo e que leve em consideração a relevância e o desafio da implementação das ações intersetoriais entre os serviços de APS e o setor de educação no cuidado à saúde e na implementação das ações de prevenção e de promoção da saúde ao adolescente na escola. Sugere-se, ainda, a inclusão de outras questões no inquérito da PeNSE, permitindo o conhecimento a respeito da percepção dos adolescentes sobre a qualidade do cuidado e do desenvolvimento de ações de promoção da saúde desenvolvidas nos serviços de APS ^48^, além de questões que favoreçam uma melhor análise da variável fonte usual de cuidado.

Faz-se necessária a implementação da intersetorialidade como estratégia de políticas públicas, que promova o protagonismo desses sujeitos adolescentes no cuidado à saúde. Portanto, as ações compartilhadas entre educação e saúde precisam ser planejadas como uma prática inovadora e integrada, com implementação por meio de uma equipe multidisciplinar, com atuação interdependente, considerando os determinantes sociais dos indivíduos. A ação intersetorial precisa ser planejada e incluída na prática dos profissionais, possibilitando, assim, a construção de saberes dialógicos e contextuais, além da interação e da construção de vínculo entre adolescentes, profissionais e instituições-serviços.
